# The effects of a post-exercise carbohydrate and protein supplement on repeat performance, serum chemistry, insulin and glucagon in competitive weight-pulling dogs

**DOI:** 10.1017/jns.2017.23

**Published:** 2017-06-05

**Authors:** Christopher W. Frye, Gretchen M. VanDeventer, Gina K. Dinallo, Jennifer A. Poplarski, Sabine Mann, Ella Pittman, Brian M. Zanghi, Joseph J. Wakshlag

**Affiliations:** 1 Department of Clinical Sciences, Cornell University College of Veterinary Medicine, Ithaca, NY 14850, USA; 2 Nestlé Purina Research, St. Louis, MO, USA

**Keywords:** Athletic dogs, Muscle, Maltodextrin, Exercise, Protein supplements

## Abstract

The physiological demands of weight-pulling dogs have yet to be investigated. Two groups of competitive weight-pulling dogs both underwent two identical pull series 3 h apart. The control group (*n* 8) was compared with a group fed a rapidly digestible carbohydrate and protein supplement after the first pull series (*n* 9). Blood was drawn before and after each pull series as well as at 15 and 30 min after the first pull series finished. Biochemistry values remained unremarkable throughout the study in both groups regardless of supplementation or exercise over time. Lactic acid showed mild significant increases post-exercise (2·1 (sd 1·2) mmol/l) compared with baseline (1·4 (sd 0·3) mmol/l; *P* = 0·03) after the initial pull series. When examining the effects of time there was a significant increase in insulin from baseline (median of 10·8 (range 6·8–17·4) μIU/ml) compared with 30 min after supplementation (17·0 (range 8·1–33·0) μIU/ml) and at 3 h after supplementation (19·2 (range 9·7–53·4) μIU/ml). In the treatment group there was also a time effect, with glucagon being elevated from baseline (median of 100 (range 79–115) pg/ml) compared with 30 min after supplementation (114 (range 90–183) pg/ml) and after the second pull series (131 (range 107–152) pg/ml). Evaluation of each dog's ability to pull the same or greater amount of weight on the second pull series revealed no significant differences. In conclusion, weight-pulling dogs have mild elevations in lactate reflecting little anaerobic metabolism compared with other canine sprinting athletes; hormonal changes associated with carbohydrate absorption are reflected within the treatment group, and supplementation had no effect on performance.

The metabolic demands of both sprinting and long-distance canine athletes have been investigated^(^^1^^–^^5^^)^; however, those of weight-pulling dogs remain unknown. The nature of many canine activities and their venues preclude a controlled competition to attain meaningful measurements to assess dietary supplementation effects on repeat performance. The canine sport that might enable the observation of muscle fatigue in the most predictable fashion, while simultaneously evaluating muscle strength, is repeated weight pulling.

Traditionally, the dog has been viewed as an endurance athlete whose energy is primarily based on lipid metabolism^(^^1^^–^^3^^)^. However, newer information suggests that even endurance dogs use significant amounts of glucose as fuel during exercise^(^^4^^)^. In prior studies, sled dogs^(^^1^^,^^2^^)^ and greyhounds^(^^5^^)^ have been shown to exhibit a decrease in skeletal muscle glycogen during racing. Furthermore, maltodextrin supplementation to sled dogs after exercise improves glycogen repletion, highlighting muscle glycogen use in canine athletes^(^^2^^,^^3^^,^^6^^)^.

Catabolism of muscle protein also occurs both during and after exercise^(^^7^^)^. Oxidation of branched-chain amino acids causes a decrease in muscle protein synthesis and an increase in muscle protein degradation^(^^8^^)^. In human athletes, the leucine concentration in blood and skeletal muscles, as well as in blood of exercise-conditioned dogs (B Zanghi, unpublished results), decreases significantly following exercise^(^^9^^)^ and may contribute to muscle fatigue^(^^10^^)^. To address this, research with human subjects has demonstrated that doses of branched-chain amino acids (0·05 to 0·1 g/kg body weight) ingested before endurance and resistance exercise reduced muscle damage biomarkers (i.e. serum creatine kinase), muscle soreness during recovery and reduced muscle fatigue^(^^10^^)^. Therefore, adding protein to the post-exercise maltodextrin source may provide a synergistic effect to increase the rate of glycogen repletion^(^^11^^–^^14^^)^, as well as reduce muscle protein catabolism.

Based on this body of research, canine competitive weight pulling was hypothesised to be a reasonable model to examine muscle strength, muscle fatigue, as well as to determine the practical use of a maltodextrin/protein supplement to improve repeated exercise performance. The goals of our study were three-fold: (1) characterise the biochemistry profile of weight-pulling dogs relative to other previously described canine sports; (2) demonstrate potential changes in glucose, insulin, lactate and biochemical profiles during resistance exercise with and without a maltodextrin and protein supplement; and (3) determine whether dietary supplementation improved performance in repeat bouts of exercise.

## Materials and methods

### Population

Competitive weight-pulling dogs (*n* 17) were privately owned and were enrolled only after owner consent had been obtained from a Cornell University Institutional Animal Care and Use Committee-approved protocol. Dogs were dichotomised into two groups by coin flip: one was provided a carbohydrate and protein supplement after an initial bout (pull series) of exercise and the other was not. Before the study, the dogs were rested at least 1 week from previous competition to limit potential fatigue. The diet of the study candidates was not altered; however, the dogs were fasted 6 h prior to competition. The ages ranged from 2 to 7 years and all were sexually intact. The control group consisted of two Alaskan huskies (two males), three pitbull/mastiff crosses (two males/one female), one Belgian shepherd (male) and two Malamutes (one male/one female), while the treatment group consisted of three Alaskan huskies (two males/one female), two pitbull/mastiff crosses (two males), one Siberian husky (male) and three Malamutes (one male/two females). Body weights ranged from 22 to 48 kg and the body condition score of the competitors ranged between 4 and 5 out of 9.

### Dietary supplement

The dietary supplement consisted of maltodextrin–dextrose (5 % dextrose, 32·4 % maltodextrin) and whey/soya protein (16 %) and was provided as recommended on the packaging for body-weight range (1·5 g/lb (3·3 g/kg) body weight, Purina ProPlan ReFuel Nutritional Supplement Bar; Nestlé Purina). The non-treated group was provided no supplement, and both groups were provided water immediately after the event and again before completing the second pull series.

### Exercise design

The weight pulls utilised wheeled carts with removable cement blocks over a concrete slab covered by short carpet that is routinely used for training and competition. The dogs began at 50 % of their historically maximum weight pull. Weight was added in accordance with the handler to simulate a competitive pull over a standard distance (16 feet; 4·88 m). All dogs participated voluntarily and were motivated by positive encouragement from their handler. Maximal weight pulled was the outcome measure with each dog having 1 min to complete the pull. Up to eleven total pulls were accomplished (range 7–11) and the pull weight was recorded for the initial pull series. The pull weight was then replicated in the second series to determine if the dog would pull equal or more weight *v.* less weight on the second attempt. The dogs were rested for 180 min between the first and second pull series, in which they received either the dietary supplement immediately after the initial pull series or no supplement and allowed to rest quietly.

### Sampling

Six separate blood collections were performed (5 ml each draw) pre-exercise to establish a baseline and at various intervals after the initial pull-series (0, 15, 30 and 180 min post-exercise), with a final sample upon completion of the second series of pulls (post-no. 2 pull). The 180 min post-exercise sample was collected immediately before starting the second pull (pre-no. 2 pull). Routine serum chemistry panel was performed before and immediately after both weight pull series at the Cornell University Clinical Pathology Laboratory using a Hitachi 911 serum chemistry analyser (Hitachi-Roche Inc.). Lactate was measured at baseline and immediately after the first pull series using a Lactate Pro hand-held analyser (HAB International Ltd). Glucose, insulin and glucagon were measured for all blood draws, with hormone assays performed by the Cornell University Diagnostic Endocrinology Unit utilising canine validated human radio-immunoassays (Millipore Inc.).

### Statistics

All serum chemistry and hormone concentrations were evaluated for normality using Shapiro–Wilk tests. Log transformation was performed for all non-normally distributed data before interpretation. A repeated-measures ANOVA with the fixed effects of time and treatment, as well as their interaction and a repeated statement for time, controlling for the effects of dog and handler by including them as random effects was utilised to assess all parameters. Tukey's *post hoc* analysis was used for determine differences across time and treatment. For the concentration of lactic acid, normalisation of the data was unsuccessful and it was analysed via Wilcoxon signed-rank testing before and after the first pulling event. Successful repeat or increased weight pulled *v.* decreased weight pulled on the second event was dichotomised and analysed using a Fisher's exact test to determine a difference between groups. Significance was set at an α of 0·05 for statistical testing.

## Results

### Serum biochemistry

Lactic acid significantly increased post-exercise (2·1 (sd 1·2) mmol/l) compared with baseline (1·4 (sd 0·3) mmol/l; *P* = 0·03). No differences between treatments were observed in serum chemistries, whereas there were significant effects of time (*P* = 0·01) for bicarbonate and K, in which 0 min post-exercise values were lower and higher, respectively, from baseline and 180 min post-exercise. Other changes in serum chemistries are described in Table 1.

**Table 1.** Serum biochemistry at the first pull series (pre-pull, 0 min post-pull), at 180 min post-pull (post-exercise) and after the second pull series (post-pull no. 2) (Mean values and standard deviations)

			Pre-pull		0 min post-pull		180 min post-pull		Post-pull no. 2		*P*		
	Reference range	Supplement	Mean	sd	Mean	sd	Mean	sd	Mean	sd	Time	Treatment	Time × treatment
Na (mEq/l)	145–153 mEq/l	Yes	148	2	148	2	148	2	148	2			
		No	148	1	149	2	148	1	148	2	0·97	0·93	0·54
K (mEq/l)	4·1–5·6 mEq/l	Yes	4·6	0·2	4·7*	0·2	4·4	0·5	4·6	0·3			
		No	4·6	0·3	4·8*	0·2	4·5	0·3	4·6	0·3	0·01	0·62	0·92
Cl (mEq/l)	105–116 mEq/l	Yes	109	2	110	3	110	1	111	3			
		No	108	2	109	2	109	2	109	1	0·67	0·39	0·07
Bicarbonate (mEq/l)	15–25 mEq/l	Yes	18	2	15*	2	18	3	16	3			
		No	19	1	16*	2	18	1	17	3	<0·01	0·63	0·87
Blood urea N (mg/dl)	10–32 mg/dl	Yes	15	4	15	4	17	3	17	3			
		No	21	8	21	8	19	6	19	6	0·95	0·15	<0·01
Creatinine (mg/dl)	0·6–1·4 mg/dl	Yes	1·1	0·2	1·1	0·2	1·0*	0·2	1·1*	0·2			
		No	1·1	0·2	1·1	0·2	1·1*	0·3	1·1*	0·2	<0·01	0·57	<0·01
Ca (mg/dl)	9·3–11·4 mg/dl	Yes	10·4	0·5	10·4	0·6	10·2*	0·5	10·1*	0·6			
		No	10·5	0·2	10·5	0·3	10·5	0·2	10·5	0·3	0·02	0·32	0·27
Phosphate (mg/dl)	2·9–5·4 mg/dl	Yes	3·7	0·7	3·6	0·6	3·4	1·1	3·3	0·9			
		No	3·9	0·7	4·0	0·7	4·0	0·7	4·0	0·7	0·65	0·22	0·27
Mg (mEq/l)	1·4–2·2 mEq/l	Yes	1·7	0·1	1·7	0·1	1·6	0·1	1·6	0·1			
		No	1·7	0·1	1·7	0·1	1·7	0·1	1·7	0·1	0·14	0·29	0·19
Total protein (g/dl)	5·3–7·0 g/dl	Yes	6·5	0·3	6·6	0·3	6·4*	0·3	6·3*	0·4			
		No	6·7	0·5	6·8	0·5	6·6	0·4	6·7	0·5	<0·01	0·24	0·06
Albumin (g/dl)	3·1–4·2 g/dl	Yes	3·7	0·2	3·7	0·2	3·6*	0·2	3·6*	0·2			
		No	3·7	0·2	3·8	0·2	3·7	0·3	3·7	0·2	<0·01	0·61	0·36
Globulin (g/dl)	1·9–3·6 g/dl	Yes	2·9	0·3	2·9	0·4	2·8	0·4	2·7	0·3			
		No	3·0	0·6	3·0	0·7	2·9	0·6	3·0	0·6	0·25	0·49	0·27
Alanine transaminase (U/l)	20–98 U/l	Yes	66	94	67	97	65	90	65	89			
		No	57	47	58	46	56	44	58	44	0·27	0·82	0·71
Aspartate transaminase (U/l)	14–51 U/l	Yes	27	8	31	12	31	7	31	6			
		No	25	7	26	9	28	8	31	10	0·03	0·55	0·06
Alkaline phosphatase (U/l)	17–111 U/l	Yes	28	12	27	11	27	11	28	11			
		No	28	10	28	10	27	9	27	10	0·43	0·95	0·31
Creatine kinase (U/l)	48–261 U/l	Yes	104	21	107	57	115	24	115	36			
		No	101	47	102	53	113	63	136	79	0·16	0·95	0·61
C-reactive protein (mg/dl)	Not available	Yes	4·6	5·2	4·8	5·5	4·7	5·9	4·8	5·9			
		No	2·9	2·1	2·7	2·0	2·7	1·9	3·0	1·9	0·73	0·40	0·67

* Mean value was significantly different from that at baseline (pre-pull time point) (*P* < 0·05).

### Serum insulin and glucagon

A significant time × treatment interaction (*P* < 0·01) was observed for both insulin and glucagon concentrations, in which dogs in the supplemented group had higher serum insulin concentrations compared with the control group at 30 and 180 min post-exercise (*P* < 0·05). By contrast, glucagon concentration was elevated from baseline at 30 min post-exercise and immediately after the second pull in the treatment group. In addition, glucagon concentration also differed (*P* < 0·05) between the two treatment groups after the second weight pull (180 min post-exercise) (Table 2).

**Table 2.** Serum glucagon, insulin and glucose at the first pull series (pre-pull, 0 min post-pull), at 15, 30 and 180 min post-pull (post-exercise) and after the second pull series (post-pull no. 2) (Mean values and standard deviations; medians and ranges)

			Pre-pull		0 min post-pull		15 min post-pull		30 min post-pull		180 min post-pull		Post-pull no. 2		*P*		
	Reference range	Supplement	Median	Range	Median	Range	Median	Range	Median	Range	Median	Range	Median	Range	Time	Treatment	Time × treatment
Insulin (μIU/ml)	Not available	Yes	10·8	6·8–17·4	9·6	6·5–13·7	12·3	7·2–19·3	17·0*†	8·1–33·0	19·2*†	9·7–53·4	11·2	6·5–22·1			
		No	11·2	5·8–16·6	10·5	5·5–13·4	8·8	4·8–13·1	7·1	5·0–15·8	7·8	5·5–40·0	8·7	6·4–13·6	0·06	0·02	<0·01
Glucagon (pg/ml)	Not available	Yes	100	79–115	95	79–108	110	82–155	114*	90–183	116	94–165	131*†	107–152			
		No	111	97–127	108	97–141	118	92–175	118	91–147	106	74–123	99	63–132	<0·01	0·09	<0·01
Glucose (mg/dl)‡	63–118 mg/dl	Yes	96	13	96	11	92	9	102	14	99	14	96	14			
		No	95	11	102	6	98	11	97	6	97	7	100	9	0·70	0·79	0·33

* Median value was significantly different from that at baseline (pre-pull time point) (*P* < 0·05).

† Median value was significantly different from that of the unsupplemented group at that time point (*P* < 0·05).

‡ Mean values and standard deviations.

### Exercise performance

Evaluation of each dog's ability to pull the same or greater amount of weight on the second pull series revealed that four of the eight dogs in the control group pulled equal or greater amounts of weight, whereas in the supplement group eight of the nine participants pulled an equal or greater amount of weight during the second pull series (*P* = 0·13).

## Discussion

The model used in this study closely replicated a typical repetitive weight-pulling event experienced by competitive weight-pulling dogs using wheeled carts^(^^15^^)^. Statistically, we found that dogs were able to perform as well on their second pull series regardless of post-exercise supplementation; however only one dog in the treatment group could not pull the peak amount during the second pull when compared with four of eight in the unsupplemented control group. It is possible that the lack of statistical significance could be due to the small sample size and insufficient power in this study. Previous research demonstrated that more rigorous exercise in dogs fed a supplement (identical to the one in our study) immediately after exercise had significant increases in blood glucose at 15, 30 and 60 min^(^^16^^)^. Carbohydrate supplementation has been shown to begin replenishing muscle glycogen storage in exercising canine athletes within 4 h after consumption^(^^2^^,^^3^^,^^6^^)^. Therefore, it was hypothesised that the supplement used in the present study would have time to initiate muscle glycogen repletion and proteosynthetic mechanisms, thus potentially improve performance by reversing the catabolic state during the intervening rest period.

Dog muscle is highly aerobic in its capacity, yet still relies on anaerobic breakdown of glycogen stores resulting in lactate accumulation^(^^5^^)^. Racing greyhounds exhibit lactate increases of sixteen times baseline after a typical race (around 30 mmol/l post-exercise)^(^^5^^,^^17^^)^. Changes in lactate before and after exercise have been described in search-and-rescue, agility, long-distance sled dogs and field-trial dogs as well^(^^5^^,^^18^^–^^24^^)^. To the best of our knowledge, lactate changes have never before been investigated for resistance-type canine sport. Lactate concentrations immediately after canine exercise, excluding greyhounds, have been about 2–5 mmol/l, with agility and search-and-rescue dogs coming in around the higher end of that spectrum. In fact, agility dog lactate decreased to baseline within 15 min of finishing a trial^(^^20^^)^ and greyhound lactate decreased to baseline within 30 min of the end of a race^(^^5^^)^. Our sampling occurred within 1 min of cessation of exercise and serum lactate averaged just over 2 mmol/l, suggesting that this type of exercise is not highly anaerobic in nature.

The biochemistry profile of weight-pulling dogs remained relatively unchanged with exercise or supplementation. However, the reduction in serum bicarbonate does reveal that the exercise was strenuous enough and corresponds to the observed increase in respiration rate by the dogs, which would probably cause an acute decrease in blood CO_2_ and increased blood pH similar to that observed in field-trial Labrador retrievers^(^^22^^)^. Interestingly, creatine kinase and aspartate transaminase values, which have been used as an indirect measurement of both clinical and subclinical muscle stress in dogs during exercise, showed no differences within 3 h of the initial pull series. Weight pulling as a sport does not appear to be overtly strenuous on muscle in general and values tend to be in line with observations in competing agility dogs^(^^20^^,^^21^^)^, although measurement at 24 h post-exercise may have been a better time point to see subtle changes. The electrolyte disturbances, shifts in packed cell volume, and increased total protein that is suggestive of haemoconcentration or fluid shifts observed in greyhound, sled dog and agility competitions did not occur in this cohort of weight-pulling dogs^(^^19^^–^^22^^)^.

Although exercise alone had no impact on serum glucose concentration, weight-pulling dogs fed the supplement experienced changes in insulin and glucagon compared with the control dogs. Insulin physiologically responds to elevations in postprandial serum glucose to support an anabolic state and typically decreases in response to exercise as working muscle glycogen metabolism conversely dictate blood glucose absorption^(^^12^^,^^13^^,^^25^^)^. Wakshlag *et al.*^(^^6^^)^ also demonstrated a similar mutual glucagon and insulin rise after exercise with similar dietary supplementation.

### Conclusion

Single-day repeat performance measures for a weight pulling were not different when comparing dogs provided a dietary supplement rich in rapidly digestible carbohydrates and protein after the initial pull series compared with those that were not. An insulin and glucagon response was observed within the supplemented group, possibly helping to maintain blood glucose in the face of exercise. Serum biochemistry remained relatively unchanged in regard to exercise or dietary supplementation, and lactate concentrations were only mildly elevated after exercise and comparable with that seen in agility dogs. Further investigation is warranted regarding the use of a dietary supplement on repeat performance; however, a more rigorous model of canine fatigue, possibly by assessing multiple consecutive days of exercise training, or applying more sensitive methods of examining cellular and metabolic responses to dietary supplementation may reveal other differences.
